# Editorial: Steroids and the Brain

**DOI:** 10.3389/fendo.2020.00366

**Published:** 2020-06-03

**Authors:** Takayoshi Ubuka, Vance L. Trudeau, Ishwar Parhar

**Affiliations:** ^1^Waseda Research Institute for Science and Engineering, Waseda University, Tokyo, Japan; ^2^Department of Biology, University of Ottawa, Ottawa, ON, Canada; ^3^Brain Research Institute, Monash University Malaysia, Subang Jaya, Malaysia

**Keywords:** neurosteroids, sexual dimorphism, sexual behavior, memory, neuropsychiatric disorders, Alzheimer's disease, stress, endocrine disrupting chemicals

Steroids contain the perhydrocyclopentanophenanthrene ring in their chemical nuclei. In vertebrates, steroids are synthesized in gonads, adrenal, and other endocrine glands and secreted into general circulation as hormones. Steroids and their receptors play significant roles in broad functions of the brain, such as regulation of socio-sexual behavior, aggression, neurogenesis, learning and memory, stress, cognition, mood and emotion. However, the brain is not only a target of steroids action but may also be the site of *de novo* synthesis from cholesterol or their precursors entering the brain. Malfunctions of steroid synthesis and signaling are related to a variety of human disorders such as gender dysphoria, anxiety, depression, autism spectrum disorder, and aging related diseases notably Alzheimer's, among others. Therefore, this Research Topic aimed to collect knowledge in all aspects of steroid function in the brain from an evolutionary to physiological and pathological standpoints, which may bring new insights into steroid actions. The subtopics include neurosteroids, sex steroids and sexual dimorphism, learning, memory, various neuropsychiatric disorders, stress and steroids, and endocrine disrupting chemicals.

## Neurosteroids

Neurosteroids are metabolic steroids synthesized from cholesterol in the central and the peripheral nervous systems (1, 2). The first article, a perspective of Steroids and Brain, is written by Baulieu who was the first to discover local synthesis of steroids in the brain (3). This perspective specifically reports on MAP4343, a synthetic pregnenolone-derivative. Additionally discussed is FKBP52, a key protein component of hetero-oligomeric steroid receptors, which interacts with Tau protein, thus playing important roles in Alzheimer's disease and other dementias.

The second article by Diotel et al. reviews neurosteroidogenesis and signaling of estrogen, progestogen, and androgen in the brain of fish, birds, and mammals and discusses the roles of sex steroids in neurogenesis, neuroprotection, and sexual behavior (4). This review further probes how steroids and lipoproteins are transported between the periphery and the brain. The authors emphasize the beneficial effects of steroids and lipoproteins against ischemic stroke, also highlighting their potential anti-inflammatory, antioxidant, and neuroprotective properties.

The third article by Da Fonte et al. is an original research article that reports on secretoneurin A (SNa) and regulation of goldfish radial glial cells (RGCs). The neuropeptide SNa is derived from proteolytic processing of the secretogranin-2 in magnocellular cells within the RGC rich preoptic nucleus. Radial glial cells are the main macroglia and have established roles in neuroesgtogen synthesis as the only site for aromatase B expression in the teleost brain, a characteristic linked to neurogenesis (5). Their previous study indicated that SNa inhibits the expression of aromatase B that converts estrogens from androgen in RGCs (6). The new results based on transcriptomic analysis suggest additional roles for SNa in the control of cell proliferation and neurogenesis.

The next original research article by Ulhaq and Kishida investigated the role of aromatase B (7) in the development of serotonergic neurons. Aromatase B is highly expressed during early development of the zebrafish brain. Early development of serotonergic neurons is also considered to play important roles in neurogenesis. In this article, they tested the effect of estradiol administration and morpholino mediated aromatase B knockdown in zebrafish embryos and larvae. Their results suggest that neuroestrogen synthesis sustains early development of serotonergic neurons.

Brain-derived steroids also act locally to affect socio-sexual behaviors (8) and locomotor movements associated with migration (9). Wingfield et al. discuss how and why neurosteroid production evolved and why peripherally produced steroids do not always fulfill central roles. Their investigations on free-living animals suggest that neurosteroids may have evolved to regulate specific behavior throughout the year independently of different life history stages. They highlight two examples. The first is the control of territorial aggression of songbirds in autumn by sex steroid production from circulating precursors such as dehydroepiandrosterone (DHEA) or *de novo* in the brain. The second example is the production of 7α-hydroxypregnenolone within the brain that appears to affect locomotor behavior in several contexts.

## Sex steroids and Sexual Dimorphism

Sex steroids coordinate the development and maintenance of the central nervous system. In the first article of this subtopic, Larson discusses the relationship between sex steroids and neuroinflammation, and the impact on neuropsychiatric and neurodegenerative disorders (10). She highlights the complex interactions between sex steroids, neuroinflammation, and regeneration of the central nervous system through adult neurogenesis.

Sex steroids also play key roles in the regulation of social recognition, reproductive behavior and parental care, which are highly sexually dimorphic. However, contribution of sex steroids in modulating adult neurogenesis in the forebrain ventricular-subventricular zone that continuously generates new neurons throughout life is underestimated (11). Ponti et al. review the literature describing sexual dimorphism and sexual differences across the physiological phases.

Steroids play important roles in sexually dimorphic brain development during perinatal and pubertal periods. It was previously demonstrated that estrogen receptor α and aromatase genes are essential to sexual differentiation of the anteroventral periventricular nucleus (AVPV) and the principal nucleus of the bed nucleus of the stria terminalis (BNSTp) in mammals (12, 13). Androgen receptor gene is also essential to sexual differentiation of the BNSTp. Kanaya et al. studied if these genes are sexually differentially expressed in the AVPV and BNSTp during puberty. Their results suggest that testicular testosterone may affect the formation of male BNSTp during puberty *via* androgen receptor and estrogen receptor α after conversion to estradiol by aromatase.

Cao et al. review sex differences in glutamatergic synaptic inputs and intrinsic excitability of rat medium spiny neurons, the output neurons of the striatum (14). They also review evidence for estradiol-mediated sexual differentiation in the nucleus accumbens core (15). The striatal brain regions including the caudate-putamen, nucleus accumbens core and shell are interesting because they express membrane-associated but not nuclear estrogen receptors. The authors conclude that striatal brain regions exhibit heterogeneity in sex differences in electrophysiological properties.

Although estrogens play important roles in sexual dimorphism of the brain, whether and how estrogens regulate the cerebral cortex are not fully understood. Denley et al. review evidence that estrogens regulate the molecular machinery required for fine-tuning the processes central to the cortex (16). The authors also discuss how estrogens regulate the function of the key molecules and signaling pathways involved in corticogenesis and highlight whether these processes are sexually dimorphic.

Funabashi et al. hypothesize that transsexual humans produce different gonadotropin levels in response to sex steroids stimulation, because the bed nucleus of the stria terminalis was suggested to be involved in gender identity and this brain area is involved in gonadotropin secretion (17). The authors examined if estrogen combined with progesterone leads a change in gonadotropin secretion in female-to-male, male-to-female transsexual, and control subjects. Their results suggest that the brain area related to gender identity may also be involved in gonadotropin secretion in humans.

The next original research article by Sano et al. investigated the role of estrogen receptor β in the dorsal raphe nucleus on female sexual behavior in mice. Previously, the authors showed that estrogen receptor β may have an inhibitory role in lordosis behavior of female mice (18). This study focused on the dorsal raphe nucleus that expresses estrogen receptor β in higher density than estrogen receptor α (19). Specific knock down of estrogen receptor β in the dorsal raphe nucleus showed the inhibitory role of estrogen receptor β in this nucleus on sexual behavior on the day after estrous in cycling female mice.

The last article of this subtopic discusses retinal disorders by Nuzzi et al. Epidemiological studies and research articles indicate a correlation between many retinopathies and sex due to potential effects of sex steroids against the development of certain disorders (20). For example, macular holes are more common in women than men, particularly in post-menopausal women. The course of retinitis pigmentosa appears to be ameliorated by progestin therapy. Diabetic retinopathy appears to be more common among men than women. The authors conclude that sex steroids may be useful for the treatment of eye diseases, particularly retinal disorders.

## Learning, Memory, and Various Neuropsychiatric Effects

Original research by Jakob et al. concerns the interactions of estrogen and the genotype of dopamine transporter (DAT1) in reinforcement learning in humans (21). The authors assessed how the natural rise of 17β-estradiol (E2) in the late follicular phase and the 40 base-pair variable number tandem repeat polymorphism of DAT1 affects reinforcement learning capacity. Their data suggest an interaction of DAT1 genotype and the transient hormonal state. They found that carriers of the 9-repeat allele experienced a significant decrease from early to late follicular phase in the ability to avoid punishment.

Ratner et al. extend their discovery of positive and negative neurosteroid allosteric modulators of GABA type-A, NMDA, and non-NMDA type glutamate receptors (22, 23) toward a state-of-the art view of how modulation of neural circuitry may affect memory and memory deficits. They conclude that the effects of neurosteroids on neural networks across the life span of males and females point to an underlying pharmacological connectome that may modulate memory across diverse altered states of mind.

The article by Hojo and Kawato reviews the local production of sex steroids in the hippocampus, a center for learning and memory in adult rodents. Hippocampal principal neurons have a complete system for sex steroids biosynthesis in males. Another recent study from the same group clarified that the levels of hippocampal steroids fluctuate across the estrous cycle in adult female rats (24). They also introduce a direct evidence of the role of hippocampal neurosteroids in hippocampal function including neurogenesis, long-term potentiation, and memory consolidation (25).

Hippocampal sex steroids including 5α-dihydrotestosterone (DHT), testosterone (T), and E2 rapidly modulate dendritic spines, which is essential for synaptic plasticity and memory (26). Soma et al. investigated the possible involvement of Src tyrosine kinase in the rapid changes of dendritic spines in response to DHT, T, and E2 using hippocampal slices of adult male rats. DHT, T, and E2 increased the total density of spines, and differentially modified the morphology of spines. However, a Src tyrosine kinase inhibitor completely blocked the increases in spine numbers induced by these steroids, indicating that Src kinase is essentially involved in non-genomic modulation of spine density and morphology induced by sex steroids.

Domonkos et al. reviewed the effects of T on anxiety during development in rodents (27). It was found that females are less anxious than males from puberty to middle age. Early organizational effects of T may influence anxiety-like behavior of females and males. However, it may be modified by activational effects of T and its metabolites. They conclude that the effects of sex steroids leading to anxiogenesis or anxiolysis depend on factors that affect hormonal status, such as age (28).

Aggression is an essential social behavior that increases survival and reproductive fitness. Munley et al. discuss the neuroendocrine mechanism of aggression in Siberian hamsters which display robust neural, physiological, and behavioral changes across seasons. The authors showed considerable evidence that DHEA, an adrenal hormone precursor, is important in maintaining aggression during the non-breeding season both in male and female hamsters (29). They conclude that adrenal DHEA likely serves as an essential precursor for neural androgen synthesis during non-breeding season (30).

Previously, it was found that E2 replacement in ovariectomized female rats reduced seizure related damage in the sensitive hilar region of hippocampal dentate gyrus (31). Iacobas et al. determine the protective effects of E2 against kainic acid-induced status epilepticus associated transcriptome alterations in the dentate gyrus of ovariectomized female rats. Their results suggest that the estrogen signaling pathway acts like a buffer against status epilepticus induced alteration of neurotransmission, which possibly contributes to E2 mediated maintenance of brain function after status epilepticus in post-menopausal women.

Tobiansky et al. highlight how androgens alter behavioral flexibility, decision making, and risk taking in their review article. After reviewing the neuroanatomy of the mesocorticolimbic system, they present evidence that androgen and other steroid receptors are present in the mesocorticolimbic system (32). They then describe evidence for local androgen synthesis in mesocorticolimbic regions (33). This review also describes how androgens modulate the neurochemistry and structure of the mesocorticolimbic system, especially the dopaminergic system. Finally, they discuss how androgens influence executive functions.

It has been observed that pervasive age-related dysfunction in hypothalamic-pituitary-gonadal axis is associated with cognitive impairments in aging and age-related neurodegenerative diseases such as Alzheimer's disease. Although estrogen modulates cognition, the effect of estrogen replacement therapy on cognition and disease diminishes with advancing age. Bhatta et al. highlight the important role for luteinizing hormone in brain function (34, 35).

## Stress and Steroids

Pinna explains that the endocannabinoid system and the biosynthesis of neuroactive steroids are involved in the neuropathology of post-traumatic stress disorder (PTSD) and major depressive disorders. The author suggests that establishing a biomarker axis for PTSD is useful to define the disorder (36). Allopregnanolone biosynthesis is downregulated in PTSD patients and stimulation of neurosteroidogenesis may be a useful strategy to treat PTSD. The author claims that peroxisome-proliferator activated receptor-α can be a target of the endocannabinoid system to enhance neurosteroidogenesis.

The article by Frost et al. is an original research on the effect of childhood emotional abuse on the associations of corticomotor white matter structure and stress neuromodulators in women with and without depression. Although experience of adversity alters the activity of the sympathetic nervous system and the hypothalamic-pituitary-adrenal axis, the underlying neural pathways are not understood well (37). The authors investigated 74 women who exhibit depression severity and/or childhood emotional abuse. They used diffusion tensor imaging to examine if the structure of white matter predicts differences in the interaction of the sympathetic nervous system and the hypothalamic-pituitary-adrenal axis as a function of early adversity. Their findings suggest that corticomotor projections may be a key to altered neural circuitry in adults with history of childhood emotional abuse.

van Campen et al. studied if stress and corticosteroids aggravate morphological changes in the dentate gyrus of the hippocampus after early-life febrile seizures in mice. It is suggested that stress is a seizure precipitant in patients with epilepsy (38). The authors investigated the consequences of ear corticosteroid exposure for epileptogenesis in mice. They investigated structural and functional plasticity in the dentate gyrus, such as changes in neurogenesis, morphology, mossy fiber sprouting, glutamatergic postsynaptic currents, and long-term potentiation. The results show that corticosterone exposure during early epileptogenesis elicited by experimental febrile seizures aggravates morphological but not functional changes in dentate gyrus.

Metabolism of glucocorticoids occurs in the brain by the actions of 11β-hydroxysteroid dehydrogenases (11β-HSD1, 11β-HSD2) (39). Rensel et al. measured 11β-HSD1, 11β-HSD2, glucocorticoid, and mineralocorticoid receptor (GR, MR) expressions in the songbird brain. 11β-HSD2, GR, and MR mRNAs were expressed throughout the adult brain. 11β-HSD2 expression covaried with GR and MR mRNAs in several brain regions. Although 11β-HSD1 mRNA was undetectable in the adult brain, the brain of developing bird expressed low levels of 11β-HSD1 mRNA. These results suggest that 11β-HSD2 protects the adult songbird brain by rapid metabolism of glucocorticoids.

## Endocrine Disrupting Chemicals

Bisphenol A (BPA) is a xenoestrogen, which is widely used in plastic products and considered an environmental endocrine disruptor. It is thought that BPA affects normal brain development by interfering with neuronal differentiation because steroids play significant roles in brain development. Fujiwara et al. investigated the effects of BPA and bisphenol F [BPF, (40)], an alternative chemical of BPA, on neural differentiation using a human fetus-derived neural progenitor cell-line. Their results showed that BPA but not BPF decreased β III-tubulin mRNA and β III-tubulin, suggesting that BPA potentially disrupts human brain development.

The last article by Ubuka et al. searched for BPA responsive genes in the rat brain to understand modifications to neurodevelopmental processes and behavior in later life. They used transgenic rats carrying enhanced green fluorescent protein tagged to gonadotropin-inhibitory hormone (GnIH) promotor (41). GnIH is a hypothalamic neuropeptide that has inhibitory effects on gonadotropin secretion and behavior (42, 43). They found upregulation of transmembrane protease serine 2 (Tmprss2) and downregulation of Forkhead box A1. Tmprss2 immunoreactivity was observed in 26.5% of GnIH neurons in the hypothalamus of 3-day-old male rat. Their results suggest that BPA disturbs the neurodevelopmental process and behavior by modifying Tmprss2 and Foxa1 expressions in the brain.

## Author Contributions

TU wrote the manuscript. VT and IP edited the manuscript.

## Conflict of Interest

The authors declare that the research was conducted in the absence of any commercial or financial relationships that could be construed as a potential conflict of interest.
